# Letter to the Editor regarding “Effect of type I collagen on TLR-3 induced MMP-13 expression in human periodontal ligament fibroblasts”

**DOI:** 10.1016/j.jobcr.2026.101440

**Published:** 2026-06-10

**Authors:** Kazuhide Takada, Satoshi Hayakawa, Shihoko Komine-Aizawa

**Affiliations:** Division of Microbiology, Department of Pathology and Microbiology, Nihon University School of Medicine, Tokyo, Japan

Dear Prof. Vidya Rattan,

We write to you regarding the recently published article by Namwad et al. titled “Effect of type I collagen on TLR-3 induced MMP-13 expression in human periodontal ligament fibroblasts” in the Journal of Oral Biology and Craniofacial Research.^1^ While we found the authors' work on the inhibitory effect of poly I:C on fibroblast migration to be insightful, we feel compelled to clarify a significant misrepresentation of our own previous work within their manuscript.

In the Discussion section of their article, the authors cite our paper^2^ as reference 40 to support the statement that “poly I:C can impair wound healing with excessive MMP-13 expression.”

However, this citation is factually inaccurate in two critical aspects. First and foremost, our data explicitly demonstrated that poly I:C promotes the migration of keratinocytes and can promote wound healing, in line with a previous clinical study.^3^ This is the exact opposite of the authors' claim. Second, our study did not examine MMP-13 expression at all. While it is not surprising that the effect of poly I:C may differ depending on the cell type (e.g., keratinocytes vs. fibroblasts), citing our work as evidence of impaired wound healing is inappropriate. We believe that a thoughtful discussion addressing these cell-type-specific differences would be highly desirable for the field.

We are concerned that this miscitation may lead to confusion among the readers of the Journal of Oral Biology and Craniofacial Research and propagate a misunderstanding within the field of wound healing.

We kindly request that this clarification be considered for publication as a Letter to the Editor, or that an appropriate erratum/corrigendum be issued by the authors to ensure the scientific accuracy of the literature.

Thank you for your time and for maintaining the high standards of the journal.

## Informed consent

Not applicable, as no human subjects were involved in the preparation of this correspondence.

## Ethical approval

This article is a Letter to the Editor discussing a previously published paper. It does not contain any new studies or experiments with human participants or animals performed by any of the authors. Therefore, ethical approval was not required.

## Declaration of competing interest

The authors declare that they have no known competing financial interests or personal relationships that could have appeared to influence the work reported in this paper.
